# Rare Presentation of Refractory Thrombotic Thrombocytopenic Purpura: Jejunal Stricture

**Published:** 2017-10-01

**Authors:** Prabath K. Abeysundara, Inoshi Athukorala, K.P.C. Dalpatadu, Karthiha Balendran, M.D.S.A. Dilrukshi, GMO Fernando

**Affiliations:** 1 Postgraduate Institute of Medicine, University of Colombo, Colombo, Sri Lanka; 2 Faculty of Medicine, University of Colombo, Colombo, Sri Lanka

**Keywords:** TTP, Chron’s-disease

## Abstract

Thrombotic thrombocytopenic purpura is a rare thrombotic disease characterized by episodes of thrombocytopenia and microangiopathic hemolytic anemia due to disseminated microvascular thrombosis. Thrombotic thrombocytopenic purpura was first described in 1924 by Moschowitz as a disease presenting with a pentad of signs and symptoms (anemia, thrombocytopenia, fever, hemiparesis and hematuria). Previous studies have described atypical manifestations of thrombotic thrombocytopenic purpura such as hemolysis, anemia and thrombosis.

## Introduction

 Thrombotic thrombocytopenic purpura is a rare thrombotic disease characterized by episodes of thrombocytopenia and microangiopathic hemolytic anemia due to disseminated microvascular thrombosis. Thrombotic thrombocytopenic purpura was first described in 1924 by Moschowitz as a disease presenting with a pentad of signs and symptoms (anemia, thrombocytopenia, fever, hemiparesis and hematuria)^1^. Previous studies have described atypical manifestations of thrombotic thrombocytopenic purpura such as hemolysis, anemia and thrombosis^2^.

## Case summary

**Figure 1:**
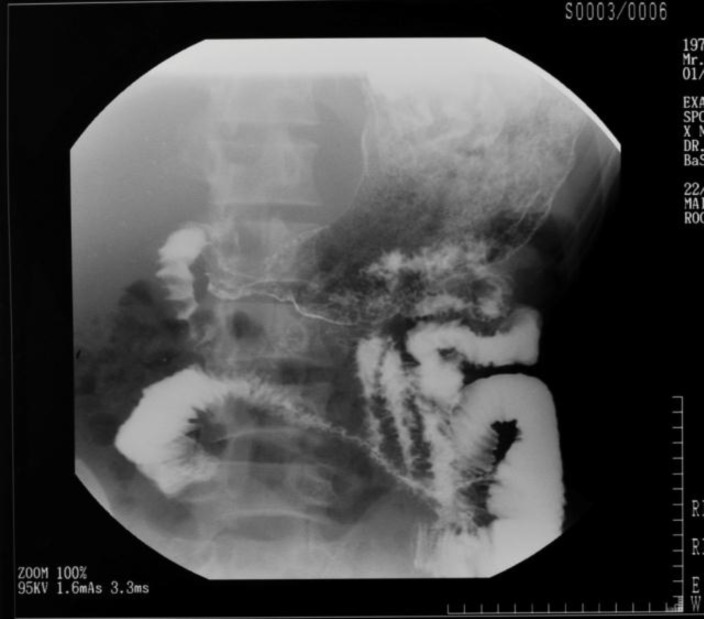
Barium meal follow-through showed an edematous stricture of the distal jejunum strongly suggestive of Crohn’s disease.

A 28-year- old man presented with a one-month history of colicky abdominal pain, hematemesis, melena, fever and watery diarrhea. There was no past medical history of note and he was employed as a rifleman in the army. Diarrhea and fever lasted 6 days and developed a generalized tonic-clonic convulsion terminated with intravenous midazolam. On examination, his body mass index was 16 Kg/m^2^. The patient was dehydrated, pale, and had mild tinge of icterus, dependent edema and moderate amount of ascites. Initial investigations showed thrombocytopenia (36 x 10^9^/L), low hemoglobin of 8 g/dl and neutrophil leucocytosis (14 x 10^9^/L). Blood film showed crenated red cells, burr cells, schistocytes, nucleated red cells and thrombocytopenia consistent with thrombotic microangiopathy. Lactate dehydrogenase level was 810 U/L. Bone marrow trephine biopsy revealed prominent megakaryopoiesis. Coagulation profile was normal. Brain magnetic resonance imaging (MRI) and electroencephalography (EEG) results were also normal. There was increased protein excretion (1.5 grams per 24 hours) and red blood cells without any casts in urine. The rate of glomerular filtration was normal. Concomitantly, inflammatory markers (erythrocyte sedimentation rate, C-reactive protein) and autoimmune profile (anti-nuclear antibody, anti- dsDNA antibody, anti- Smith antibody) remained normal. Erect abdominal radiograph showed dilated small bowel loops and multiple fluid levels. Ultrasound scan of the abdomen and contrast-enhanced computed tomography revealed mural thickening of the distal ileum, caecum, and proximal ascending colon together with moderate ascites without any evidence of thoracic or abdominal lymphadenopathy. Peritoneal fluid aspirate was pale yellow, protein: 4.2 g/dl, pus cells: 12-15/ high-power field, ADA: 16 U/L, tuberculosis PCR: negative. Cytology showed acute and chronic inflammation with epithelioid histiocyte-like cells. Mycobacterium tuberculosis and atypical mycobacterial cultures, tuberculosis interferon- gamma in serum, pyogenic culture, anti-Yersinia antibody in serum were negative. Barium meal follow-through showed an edematous stricture of the distal jejunum strongly suggestive of Crohn’s disease (Figure 1). Upper gastrointestinal endoscopy showed inflamed mucosa extending from mid esophagus, stomach and beyond second part of the duodenum. Colonoscopy revealed inflamed caecum, ileum and proximal ascending colon. Multiple biopsies revealed non-specific inflammation in hematoxylin and eosin stain. Fungal staining and periodic acid shift staining did not reveal any abnormality. Diagnostic laparoscopy showed the inflamed appendix and appendectomy was done. Histology revealed chronic inflammation and a thrombus in an arteriole without any evidence of vasculitis. Tuberculosis culture of the appendicular biopsy was negative. 

On day 27 of his hospital admission, the diagnosis of thrombotic thrombocytopenic purpura (TTP) was made. 

Oral prednisolone 1mg/Kg and daily plasma exchange of 1.5 volumes were begun. After 6 cycles of plasma exchange, platelets began to rise with disappearance of fragmented red blood cells in blood film. Diarrhea and fever settled and the patient clinically improved. After 24 cycles of plasma exchange, platelet count was 160 x 10^9^/L, and thereafter exchanges were done every other day, but reducing trend of platelets and reappearance of gastrointestinal symptoms were observed thereafter. The diagnosis of refractory thrombotic thrombocytopenic purpura was made and the patient was started on intravenous anti-CD 20 monoclonal antibodies (Rituximab) at 375/1.73 m^2^ per week for 4 weeks. Prednisolone was tailed off over one month and plasma exchange was withheld. After 3 months of Rituximab therapy, the patient was asymptomatic and platelet count remained stable around 200 x 10^9^/L. 

## Discussion

 Amorosi et al. described pentad of features of TTP including fever, bleeding or purpura generally with thrombocytopenia, microangiopathic haemolytic anaemia, neurological manifestations, and renal disease which were demonstrated in this patient^3^. Radiological finding supported the diagnosis of Crohn’s disease. But classical endoscopic features of cobblestoning, discontinuous lesions and histological features of granulomas and neuromatous lesions were not found^4^^,^^5^. Although association between Crohn’s disease and thrombotic thrombocytopenic purpura was reported, our patient did not demonstrate convincing evidence for Crohn’s disease^6^.

Gastrointestinal ischemia as a manifestation of thrombotic thrombocytopenic purpura was first reported in 1989 and other atypical manifestations are acute respiratory distress syndrome, pancreatitis, hepatitis, peripheral digital ischemia and non-occlusive mesenteric ischemia^7^. Abdominal pain, which is not considered as a classic feature of TTP, represents intra-abdominal ischemia as it has been rarely described^8^. There were few reported deaths in patients diagnosed with primary idiopathic TTP and massive gastrointestinal ischemia as well as hemorrhage confirmed by autopsy^9^. Our patient had evidence of intestinal hemorrhage and stricturing disease of the small intestine mimicking crohn's disease* at presentation.* But appearance of neurological manifestations and hematological findings led to the diagnostic conclusion of intestinal manifestations of TTP. The post infectious etiology of the disease is discussed in previous case reports^6^. The presence of chronic inflammatory cells with epithelioid histiocyte-like cells in peritoneal fluid may support chronic bacterial or fungal infection which may trigger TTP. But histological findings, fungal staining, microbiological culture or serology did not reveal any microbial etiology. 

Pathogenesis of TTP is described as severe deficiency of ADAMTS13 (< 10% activity). Acquired TTP is due to formation of anti-ADAMTS13 antibodies. Although neurologic symptoms are considered classical TTP, they are not present in all patients. Any delay in referring the patient for therapeutic plasma exchange is potentially fatal. Therefore, vigilant clinical assessment as well as early suspicion and diagnosis with great concern to atypical manifestations of TTP will reduce mortality due to the disease. 

## CONCLUSION

 Abdominal pain is a rare manifestation of TTP and when present with other manifestations of the disease, may represent micro vascular thrombosis of intra-abdominal organs.
